# Genetic Diversity and Population Structure of Shanlan Upland Rice Germplasm Based on SSR Markers

**DOI:** 10.3390/plants14203233

**Published:** 2025-10-21

**Authors:** Linan Zhai, Mingchao Zhao, Xiaowei Yan, Yapeng Li, Xiaorong Xiao, Qingyu Wang, Huijian Wang, Bangji Zhou, Yong Yun, Funeng Xing, Qingjie Tang

**Affiliations:** 1 Cereal Crops Institute, Hainan Academy of Agricultural Sciences, Key Laboratory of Crop Genetics and Breeding of Hainan Province, Haikou 571100, China; zhailn868@163.com (L.Z.); 123zhaomingchao@163.com (M.Z.); 13078994838@163.com (X.Y.); liyapeng2116@163.com (Y.L.); xiaorong1990829@163.com (X.X.); 18789169355@163.com (Q.W.); 13016296221@126.com (H.W.); 12102159@chnenergy.com.cn (B.Z.); 2 Sanya Institute, Hainan Academy of Agricultural Sciences, Sanya 572025, China; yunyong@hnaas.org.cn (Y.Y.); xingfuneng@hnaas.org.cn (F.X.)

**Keywords:** shanlan upland rice, SSR markers, genetic diversity, population structure

## Abstract

Shanlan upland rice is a unique rice resource of the Li and Miao ethnic group in China and serves as a valuable gene pool adapted to tropical mountainous environments. To explore the genetic relationships of Shanlan upland rice from different geographical origins, 21 SSR markers were used to conduct genetic diversity and population structure analyses on 288 Shanlan upland rice accessions from 10 provinces (regions) in southern China. Results: The study revealed that the mean values of effective allele number (Ne), Shannon’s information index (I), polymorphic information content (PIC), observed heterozygosity (Ho), and expected heterozygosity (He) for Shanlan upland rice were 1.616, 0.491, 0.74, 0.129, and 0.306, respectively. Genetic diversity analysis and molecular variance analysis (AMOVA) showed that the main source of variation between materials was the individual Shanlan upland rice plants. Genetic distance and differentiation results revealed the phylogenetic relationships among Shanlan upland rice populations. Both clustering and population structure analyses divided the materials into five subgroups, suggesting that the Shanlan upland rice from Qiongzhong, Hainan, might be the center of genetic diversity for the Hainan Shanlan upland rice, while rice from Dongfang, Hainan, and the inland populations exhibit genetic isolation. This study provides foundational data for the prioritized conservation and innovative utilization of Shanlan upland rice germplasm resources.

## 1. Introduction

Shanlan upland rice (*Oryza sativa* L.) is a distinctive dryland rice germplasm endemic to Hainan Province, China, carrying rich genetic diversity and deep cultural value. Through over 2000 years of agricultural practices by the Li ethnic group, this germplasm has developed characteristics such as a short growing period, low water requirements, drought tolerance, and resistance to poor soil [1,2], making it a valuable gene pool adapted to tropical mountain environments. Shanlan upland rice is primarily distributed in the mountainous regions of central and western Hainan China, including Qiongzhong, Baisha, and Ledong in China. Shanlan upland rice comes in various varieties, including red rice, black rice, fragrant rice, black spike rice, red spike rice, glutinous rice, and common rice. However, most of the Shanlan upland rice cultivated in these regions consists of seeds saved by farmers, leading to issues of genetic admixture and low yields and easy to fall down in case of typhoon and rainstorm [3].

Moreover, as Hainan is developing its specialty agriculture, the planting area of Shanlan upland rice is decreasing year by year. Despite these issues, Shanlan upland rice is a “living fossil” of Li culture, a “master” of drought resistance and poverty tolerance, and a “key” to future breeding. It has a unique taste and flavor, and is also suitable for brewing. Against the backdrop of global climate change and increasing food security challenges, studying the genetic diversity of Shanlan upland rice is crucial for ensuring food security, promoting sustainable agriculture, and protecting biodiversity.

Molecular markers, which are unaffected by plant growth stages or environmental conditions, offer significant advantages over phenotypic markers for assessing genetic diversity in plants [4]. Li et al. [5] utilized 12 SSR markers to investigate the genetic diversity of 214 drought-resistant rice varieties from Southeast Asia and five provinces in southern China. Their finding indicated that the majority of genetic variation was attributed to differences among individual rice plants, and suggested that Shanlan upland rice in Hainan may have originated from dry rice varieties in Guangdong province. Vasumathy et al. [6] revealed a high level of genetic diversity among the unexploited rice landraces cultivated by the farmers of Kerala. Nachimuthu et al. [7] used 61 genome-wide SSR markers explained that 14% of variation was due to difference between with the remaining 86% variation may be attributed by difference within groups. Similarly, Jasim et al. [8] revealed genetic diversity of aromatic rice germplasm by SSR markers. Temnykh et al. [9] used SSR markers enhanced the resolution of an existing genetic map of rice. Ali et al. employed SSR markers to assess the genetic diversity and population structure within the genera *Saccharum* (sugarcane) and *Erianthus* (wild cane) [10]. Currently, SSR markers are widely used in the genetic diversity studies of a range of crops, including cowpea [11], Sichuan pepper [12], melon [13], *Solanum* species [14], and ornamental flowers [15].

However, earlier genetic diversity studies on Hainan Shanlan rice were limited by sample sizes. For instance, Li et al. [5] analyzed only 55 samples of Hainan Shanlan upland rice, Yuan et al. [16] studied 14 samples, and Yao et al. [17] included 28 samples. To address this limitation, the Institute of Cereal Crops at the Hainan Academy of Agricultural Sciences has, since 2013, conducted an extensive survey of Shanlan upland rice cultivation across Hainan Island. This study has resulted in the collection of 288 accessions of Shanlan upland rice germplasm, including 265 samples from Hainan, 14 from Guizhou, 3 from Guangxi, 3 from Jiangxi, and 3 from Yunnan. By employing SSR molecular markers, with a larger sample size and more diverse sources than previous studies, the findings will contribute essential data for the conservation and innovative utilization of Shanlan upland rice.

## 2. Results and Analysis

### 2.1. Genetic Diversity of Shanlan Upland Rice

Genotyping of 288 Shanlan upland rice accessions with 21 SSR markers revealed a moderate level of genetic diversity (Mean Nei’s = 0.242, Mean He = 0.306, Mean PIC = 74.02%) (Table 1). The notably lower observed heterozygosity (Ho = 0.129) compared to expected heterozygosity (He = 0.306) suggests prevalent inbreeding or a structured population, which was confirmed by a high mean inbreeding coefficient (Fis = 0.513).

### 2.2. Genetic Diversity of Shanlan Upland Rice from Different Geographic Origins

Analysis of genetic diversity across ten geographical origins highlighted Qiongzhong (Hainan, China) as a region of exceptional diversity, exhibiting the highest values for the number of different alleles (Na = 2.238), effective alleles (Ne = 1.766), and Shannon’s index (I = 0.605) (Table 2). This, combined with its central location in Hainan, suggests that Qiongzhong may be a core genetic diversity center for Shanlan upland rice. In contrast, inland populations from Jiangxi and Guangxi showed the lowest genetic diversity (Na = 1.286 and 1.333, respectively; I = 0.224 and 0.230, respectively). Positive inbreeding coefficients (F) across all populations (0.200–0.633) further support the trend of inbreeding, with several populations (e.g., Yunnan, Baisha) showing F > 0.5, indicating a potential risk of inbreeding depression.

### 2.3. Nei’s Genetic Distance Among Shanlan Upland Rice Populations from Different Geographical Origin

**Figure 1:**
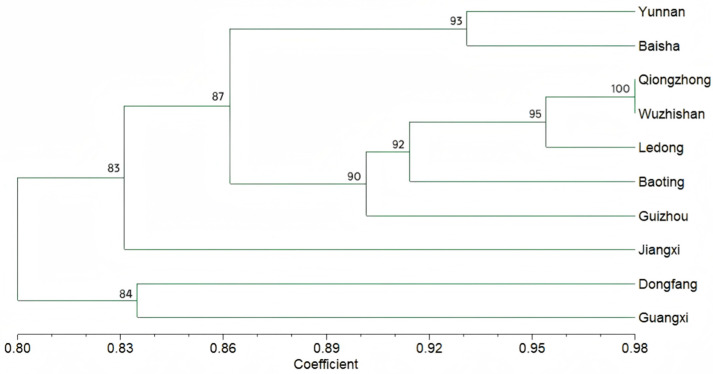
Genetic similarity between populations in different regions. The number of each branchpoint represents bootstrap values.

Population genetic analysis based on Nei’s genetic distance and Fst revealed clear genetic relationships (Table 3). According to established standards [18] populations from central Hainan (Baisha, Qiongzhong, Wuzhishan, Ledong, Baoting) formed a closely related cluster with low genetic distances (<0.05) and low Fst values (<0.1), indicating frequent gene flow. Strikingly, Dongfang (Hainan) was a notable exception, showing high genetic divergence from both other Hainan populations (e.g., Fst with Qiongzhong = 0.134) and inland populations (e.g., Nei’s distance with Guizhou = 0.345). This suggests that Dongfang might be an isolated subpopulation, possibly due to unique ecological adaptation or human practices. Inland populations (Yunnan, Guizhou, Jiangxi, Guangxi) were generally more divergent from the Hainan cluster and from each other, with the highest differentiation observed between Guangxi and Jiangxi (Fst = 0.426). A cluster analysis based on genetic similarity corroborated these findings, showing a clear separation between the main Hainan cluster, the outlier Dongfang population, and the inland populations (Figure 1).

### 2.4. Analysis of Molecular Variance

We used the AMOVA tool to explore genetic variation in 10 Shanlan upland rice populations. The results showed that 8.39% of genetic variation existed between populations and about 91.609% of genetic variation existed in 288 Shanlan upland rice germplasm (Table 4). The high within-population diversity (91.6%) indicated that each population retains a large amount of genetic variation, which was consistent with the high heterozygosity and allelic richness observed in Table 2 for most populations (except Jiangxi and Guangxi), and that reduced extinction risk from drift. The PhiPT (also known as FST) value of 0.084 was relatively low, indicating that the populations were not highly differentiated, or suggesting that there was substantial gene flow among populations or recent common ancestry. This indicates that, despite the observed population structure, each local population retains a high degree of internal genetic diversity, which is a valuable asset for conservation and breeding programs.

### 2.5. Cluster and Population Structure Analysis of Shanlan Upland Rice

**Figure 2:**
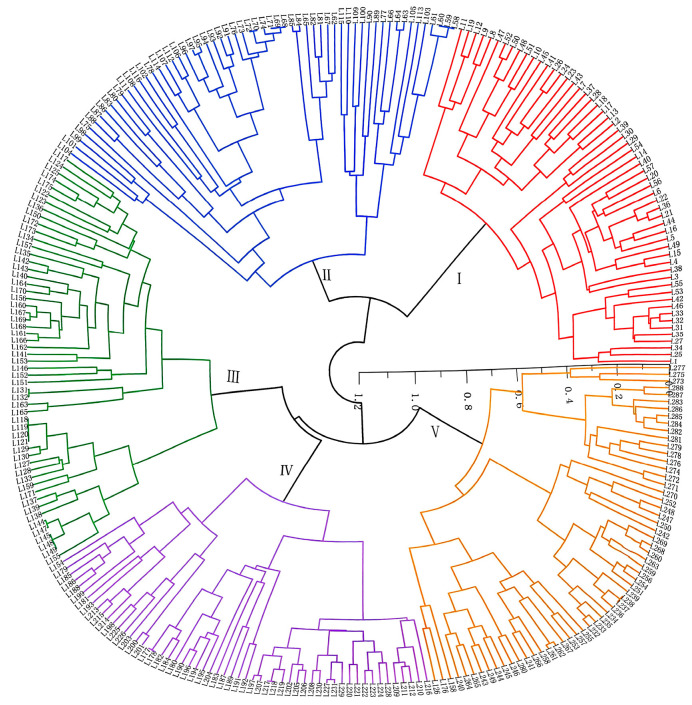
288 Shanlan upland rice (*Oryza sativa* L.) Cluster Maps. Red represents Group I, blue represents Group II, green represents Group III, purple represents Group IV, and yellow represents Group V.

**Figure 3:**
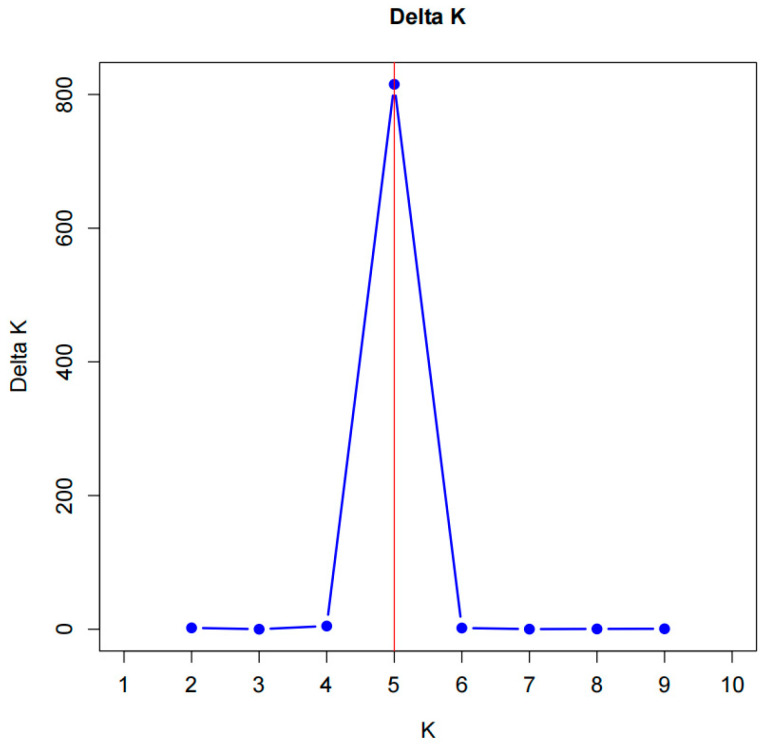
K-value and Delta K-value line chart.

**Figure 4:**
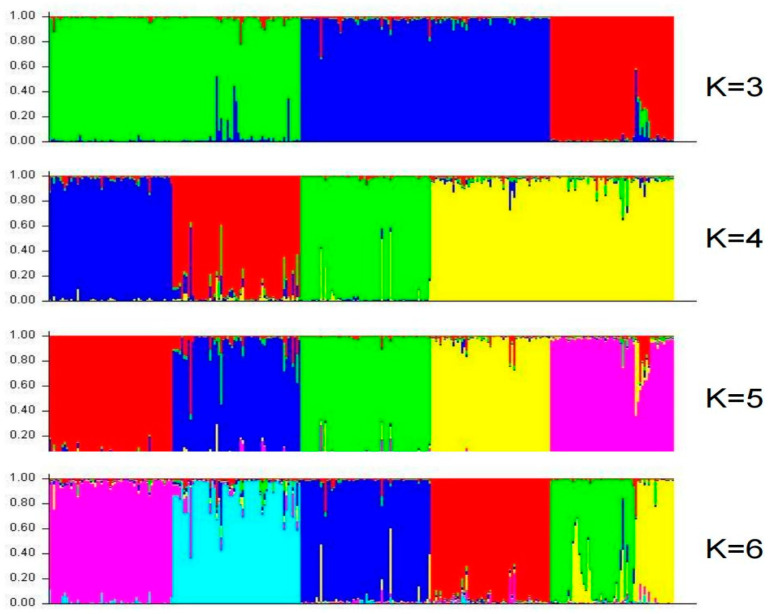
Population structure of 288 Shanlan rice varieties. Each vertical line represents one accession. When K = 5, red represents Group I, blue represents Group II, green represents Group III, yellow represents Group IV, and purple represents Group V. When K = 6, light blue represents Group VI.

Both UPGMA cluster analysis (Figure 2) and population structure analysis (Figure 3 and Figure 4) consistently grouped the 288 accessions into five distinct genetic clusters (K = 5). The distribution of accessions across these clusters strongly supports the centrality of Qiongzhong. Accessions from Qiongzhong and Wuzhishan (the most diverse regions) were distributed across all five clusters, demonstrating their rich and admixed genetic backgrounds. Conversely, accessions from low-diversity regions like Dongfang, Jiangxi, and Guangxi were confined to specific clusters, indicating genetic homogeneity.

For example: Cluster I contained 57 accessions, mainly from Baisha and Qiongzhong in Hainan, with a few from Yunnan and Wuzhishan, their commonality was that most of them were japonica rice and tended to be glutinous, which means their direct starch content was generally low. Cluster II comprised 59 accessions, predominantly from Qiongzhong, along with a few from Baisha, Wuzhishan, Ledong, and one from Guizhou, their commonality was that the altitude of the collection site was generally higher than that of the first group, and the rice of this group was mostly high-quality white rice, and some rice even has a natural aroma. Cluster III included 56 accessions mainly from Qiongzhong, Ledong, and Dongfang, with all accessions from Dongfang exclusively grouped in this cluster, their commonality was that they were more drought tolerant and mostly red rice, which may be because the Dongfang is a relatively arid place. Cluster IV consisted of 56 accessions, primarily from Wuzhishan, Qiongzhong, and Jiangxi, the outer shells of the Shanlan rice in this group were mostly colored, such as red or purple shells, but the rice was still white. Cluster V comprised 60 accessions, mainly from Guizhou, Baoting, and Guangxi, most of the Shanlan rice in this group was not glutinous, which means their direct starch content was generally high.

Based on the SSR marker data, population structure analysis of the 288 Shanlan upland rice accessions revealed a clear inflection point at K = 5, where the Delta K value was highest (Figure 3). Accordingly, the accessions were assigned to five subpopulations (Figure 4). Subpopulation I contained 57 accessions (L1–L57), mainly from Baisha and Qiongzhong, with a few from Yunnan and Wuzhishan. Subpopulation II comprised 59 accessions (L58–L116), predominantly from Qiongzhong and Ledong. Subpopulation III consisted of 60 accessions (L117–L176), mainly from Qiongzhong, Ledong, and Dongfang, with a few from Wuzhishan, Baoting, and Baisha. Subpopulation IV included 55 accessions (L177–L231), primarily from Jiangxi, Guizhou, Wuzhishan, and Qiongzhong. Subpopulation V comprised 57 accessions (L232–L288), mainly from Guangxi, Baoting, Guizhou, and Wuzhishan (Figure 4). The population structure results were highly consistent with the clustering patterns shown in Figure 2. Interestingly, some germplasm accessions showed no introgression under any K value, suggesting they may represent key ancestral types. For example, several accessions from Yunnan, Baisha, and especially Qiongzhong (up to 80 accessions) exhibited this pattern, reinforcing the conclusion that Qiongzhong serves as a core genetic center. Moreover, the stable and isolated populations from Dongfang and Jiangxi were consistent with the previously observed high genetic differentiation in these regions.

## 3. Discussion

### 3.1. Moderate Genetic Diversity and Population Structure of Shanlan Upland Rice

Genetic diversity is a cornerstone of biodiversity, providing insights into a species’ evolutionary history and endangerment status while offering essential resources for plant breeders to broaden the genetic base of crops [19,20]. Our analysis of 288 Shanlan upland rice accessions from Hainan and adjacent regions reveals a moderate level of overall genetic diversity (mean Shannon diversity index, I = 0.491). This value is significantly lower than that reported for Hainan common wild rice (I = 0.975) [15,21], a finding consistent with previous studies [5,16,22,23], the genetic diversity within subpopulations was: wild rices > landraces > cultivars. But our research results were higher than Yang’s research [24] (I = 0.491 > I = 0.2826), and lower than Wang’s [25] and Zhang’s [26] research, this might because Wang’s rice types were more diverse. This reduction in diversity is likely a consequence of Shanlan upland rice being an upland crop that has undergone long-term directional selection under similar environmental and agronomic conditions. PIC values, which are good indicators of marker polymorphism levels, were in the range of 0.20 to 1.00, with a mean of 0.74, higher than those reported in Indonesian (0.66) [27], and lower then those reported in China (0.82) [28] rice germplasm, respectively.

Further evidence of a constrained gene pool includes the low observed heterozygosity (Ho = 0.129) compared to the expected heterozygosity (He = 0.306), indicating a high degree of self-pollination. This is corroborated by the relatively high genetic differentiation among subpopulations (for example, the Fst values of Hainan Dongfang and Qiongzhong, as well as inland populations (Yunnan, Guizhou, Jiangxi, Guangxi) and Hainan clusters were all relatively high), in agreement with several studies [27,29,30], suggesting significant inbreeding and a pronounced population structure. This genetic pattern aligns with the crop’s history: cultivated for generations by the Li ethnic minority in Hainan using traditional slash-and-burn (kan-shanlan) agriculture, farmers practiced seed selection from high-yielding plants. As a predominantly self-pollinating crop, this cultivation mode naturally maintained low heterozygosity and preserved genetic integrity, a common feature of autogamous crops [31].

### 3.2. Qiongzhong as a Potential Core Genetic Center and Patterns of Regional Differentiation

A key finding of our study is the spatially heterogeneous distribution of genetic diversity. The central-western regions of Hainan—specifically Qiongzhong, Baisha, and Wuzhishan—exhibited significantly higher diversity than peripheral areas. This area, historically the heartland of Li agriculture, appears to be the core genetic center for Shanlan upland rice, with Qiongzhong showing the highest diversity. The concentration of diversity in this core region suggests a long and continuous history of cultivation and conservation by local communities.

In contrast, populations from peripheral areas, such as those in Jiangxi and Guangxi, showed lower diversity and higher differentiation. For instance, the genetic differentiation between Jiangxi and Guangxi populations was extreme (Fst = 0.426), likely driven by geographical isolation and ecological barriers. This pattern highlights how local selection pressures and genetic drift in isolated populations can enhance inter-population differentiation over time.

### 3.3. Phylogeography and Historical Dispersal Routes

The population structure analysis provides compelling insights into the origins and dispersal of Shanlan upland rice. Our data support the hypothesis that it may have been introduced from Guangdong upland rice rather than directly domesticated from Hainan wild rice. This is consistent with archeological and ethnographic evidence linking the Li people to ancient migrations of the Baiyue people, who spread rice cultivation practices across southern China [32,33,34,35].

Interestingly, we detected low genetic differentiation between populations from the Yunnan-Guizhou Plateau and several Hainan populations (e.g., Guizhou vs. Wuzhishan Fst = 0.075; Yunnan vs. Baisha Fst = 0.076). This suggests historical gene flow between these geographically distant regions, potentially supporting a dissemination route where upland rice varieties moved from Guangdong to Hainan, with subsequent connections to the southwestern plateau [16].

### 3.4. The Genetically Distinct Dongfang Population: Implications for Conservation

A particularly notable result is the pronounced genetic isolation of the Dongfang population. It exhibited the greatest genetic divergence from other Hainan populations (genetic distance > 0.18) and even larger distances from non-Hainan populations (>0.28). The high *Fst* values (e.g., 0.165 vs. Baisha) confirm its strong differentiation. This suggests that the Dongfang population has been subject to unique evolutionary pressures or prolonged geographic isolation. Consequently, conservation strategies must prioritize maintaining the genetic distinctiveness of the Dongfang population and other highly differentiated groups, as they may harbor unique alleles critical for future breeding [36].

### 3.5. Gene Introgression: A Double-Edged Sword

Our analysis also revealed evidence of gene introgression in some accessions, likely from traditional South China *indica* landraces or varieties introduced via historical trade routes like the Maritime Silk Road. Such introgression can be a double-edged sword. On one hand, it may enhance genetic diversity and adaptive traits, as demonstrated by the introgression of drought-tolerance genes from wild rice into cultivated varieties [33]. On the other hand, it can erode the unique genetic identity of Shanlan upland rice [34]. As a cultural heritage crop, a balanced strategy is required: conserving its unique genetic signature while cautiously utilizing beneficial introgressed alleles to improve agronomic traits like yield and stress resistance.

### 3.6. Limitations and Future Perspectives

This study provides the first comprehensive phylogeographic assessment of Shanlan upland rice, yet it has limitations. The use of SSR markers, while informative, captures only part of the genomic diversity. Future work employing whole-genome resequencing or SNP arrays will more precisely detect adaptive loci and historical introgression events. Additionally, broader sampling of peripheral populations and the integration of archeological and genomic dating methods will help resolve the demographic history and domestication timeline of this crop.

Future research should focus on the following: (1) Genome-wide association studies (GWAS) to identify alleles for drought tolerance and grain quality; (2) comparative genomics to uncover unique adaptive traits; (3) population genomic modeling to disentangle the effects of selection, isolation, and introgression; and (4) implementing targeted conservation genomics strategies for unique populations like Dongfang.

## 4. Materials and Methods

### 4.1. Plant Materials

**Figure 5:**
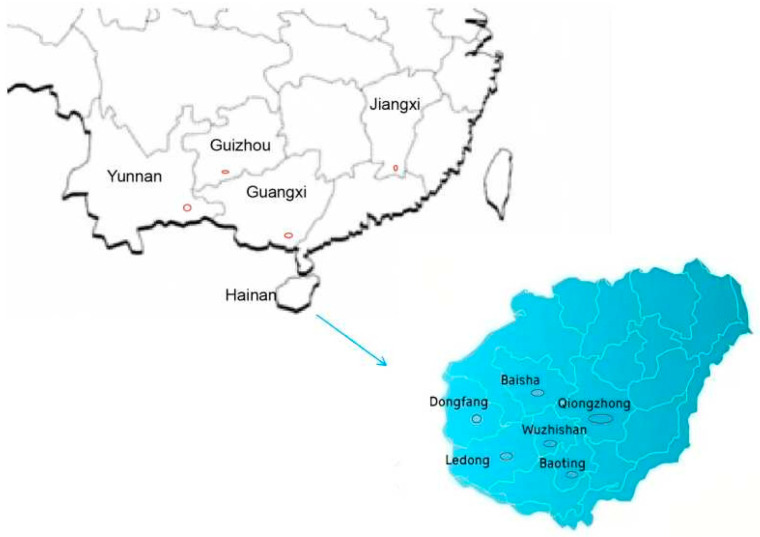
Collection sites of the ten Shanlan Upland rice populations studied.

A total of 288 Shanlan upland rice accessions were collected between 2013 and 2022 from Hainan, Guizhou, Guangxi, Jiangxi, and Yunnan provinces (Figure 5, Table 5). These samples are currently conserved at the experimental base of the Hainan Academy of Agricultural Sciences in Yongfa Town, Chengmai County, Hainan Province.

### 4.2. DNA Extraction of Shanlan Upland Rice

Fresh young leaves of Shanlan upland rice were placed in 2 mL centrifuge tubes with steel beads and flash-frozen in liquid nitrogen for one minute. The samples were then ground using a high-throughput tissue grinder. Genomic DNA was extracted using a centrifugal-column-based Plant Genomic DNA Extraction Kit (Tiangen Biotech, Beijing, China). The concentration and quality of the extracted DNA were assessed using a spectrophotometer and agarose gel electrophoresis. Finally, the DNA was diluted to 50 ng/ul, and stored at −20 °C for future use.

### 4.3. Screening and Amplification of Molecular Marker Primers

Based on published SSR markers from the rice genome and previous studies [24], 21 polymorphic primer pairs were selected for genetic diversity analysis (Table 6). These primers were screened by amplifying Shanlan upland rice accessions with distinct phenotypes and collected from geographically distant regions. The primers were synthesized by Sangon Biotech Co., Ltd. (Shanghai, China). PCR amplification was performed following the protocol described by Zhai et al. [21], with a total reaction volume of 20 μL: 4 μL DNA template (50 ng/μL), 10 μL 2× Taq PCR MasterMix II, 2 μL forward primer (10 μmol/L), 2 μL reverse primer (10 μmol/L), and 2 μL ddH_2_O. The PCR cycling conditions were as follows: initial denaturation at 95 °C for 5 min; followed by 35 cycles of denaturation at 95 °C for 30 s, annealing at 50 °C ~ 60 °C for 30 s, and extension at 72 °C for 50 s; with a final extension at 72 °C for 5 min. PCR products were separated by electrophoresis on 5% agarose gels.

Shanlan upland rice samples, sourced from various locations, include different resources such as red rice and white rice, which exhibit genomic differences in SSR loci. So, the lengths of DNA fragments obtained after PCR amplification vary among individuals. We performed PCR amplification on DNA from different samples using the same pair of SSR primers. The PCR products (i.e., DNA fragments of varying sizes) were visualized using agarose gel electrophoresis, viewed on a gel imaging system, photographed, and the electrophoresis image was saved. Subsequently, we used a marker (100–750 bp) as a reference to measure the sizes of the DNA fragments on the electrophoresis gel. The fragment sizes were recorded in an excel spreadsheet for subsequent analysis. All PCR amplifications and electrophoresis procedures were repeated three times to ensure the accuracy of the results.

### 4.4. Data Analysis

The original genotype data were transferred into GenAlEx 6.2 to calculate the various genetic diversity indicators of SSR loci and populations, the observed allele (Na), effective allele (Ne), Shannon index (I), polymorphism information index (PIC), observed heterozygosity (Ho), expected heterozygosity (He), inbreeding coefficient (Fis), molecular variance (AMOVA) were included.

Genetic similarity coefficients among Shanlan upland rice accessions were calculated using NTSYS 2.1 software. Cluster analysis was performed with the UPGMA and SHAN methods, and the resulting dendrograms were visualized using Origin 2022.

Population structure of Hainan Shanlan upland rice was analyzed using STRUCTURE 2.2 software. Using a membership probability threshold of 0.60, population K values from 1 to 10 were simulated with 5 iterations for each K using 10,000 burn-in periods followed by 100,000 Markov Chain Monte Carlo iterations in order to obtain an estimate of the most probable number of populations. Delta K was plotted against K values; the best number of clusters was determined following the method proposed by Evanno et al. [37], and obtained via the StructureSelector platform (https://lmme.ac.cn/StructureSelector/ accessed on: 26 September 2024).

## 5. Conclusions

In this study, 21 pairs of SSR markers were used to systematically analyze 288 Shanlan upland rice accessions collected from Hainan and adjacent regions. The results revealed that Shanlan upland rice exhibits a moderate level of overall genetic diversity, with population differences primarily arising from variation among individuals. Shanlan upland rice from Qiongzhong, Hainan, showed the highest genetic diversity, suggesting it may represent the core center of diversity for this species. In contrast, populations from Dongfang and several inland regions exhibited clear genetic isolation and differentiation. These findings not only elucidate the geographic genetic patterns and potential dispersal routes of Shanlan upland rice but also provide a theoretical foundation for the conservation and utilization of its germplasm resources, as well as its potential application in rice genetic improvement.

## Figures and Tables

**Table 1:** 21 pairs of primers diversity analysis.

Locus	Na	Ne	I	H	Ho	He	PIC (%)	Fis	Fit
RM7l	2.800	2.035	0.758	0.246	0.196	0.490	70.00	0.600	0.663
OSR28	2.000	1.913	0.668	0.333	0.043	0.475	93.33	0.909	0.912
RM7102	2.800	2.300	0.883	0.321	0.577	0.550	90.00	−0.050	0.130
RM571	1.400	1.114	0.118	0.070	0.096	0.075	50.00	−0.287	−0.051
RM336	1.640	1.483	0.369	0.257	0.099	0.081	64.00	−0.216	0.012
RM424	1.720	1.437	0.375	0.252	0.000	0.097	72.00	1.000	1.000
RM85	1.500	1.430	0.326	0.230	0.019	0.100	50.00	0.812	0.893
RM3331	2.000	1.857	0.652	0.454	0.058	0.459	100.00	0.873	0.880
RM567	2.000	1.875	0.656	0.145	0.783	0.464	50.00	−0.688	−0.644
RM551	1.300	1.081	0.099	0.059	0.004	0.056	30.00	0.937	0.987
RM331	2.200	1.807	0.625	0.265	0.178	0.401	85.00	0.556	0.673
RM17	2.000	1.784	0.626	0.135	0.021	0.435	100.00	0.952	0.959
RM21	1.500	1.207	0.212	0.135	0.004	0.131	50.00	0.973	0.995
RM2l9	2.000	1.718	0.580	0.397	0.004	0.397	100.00	0.991	0.992
RM190	2.000	1.495	0.493	0.237	0.045	0.319	66.67	0.859	0.894
RM231	1.200	1.021	0.139	0.019	0.200	0.100	20.00	−1.000	0.778
RM253	2.200	1.536	0.444	0.290	0.000	0.296	100.00	1.000	1.000
RM267	2.000	1.565	0.533	0.351	0.007	0.351	100.00	0.980	0.981
RM423	1.800	1.469	0.386	0.177	0.362	0.265	70.00	−0.369	0.118
RM481	2.800	2.093	0.816	0.330	0.011	0.497	93.33	0.979	0.984
RM493	2.000	1.689	0.555	0.379	0.011	0.379	100.00	0.972	0.978
mean	1.946	1.615	0.491	0.242	0.129	0.306	74.02	0.513	0.673

**Note:** Na: Differential allele. Ne: Number of effective alleles. I: Shannon Index. H: Nei’s diversity index. Ho: Observed heterozygosity. He: Expected heterozygosity. PIC: Polymorphic information content. Fis: Inbreeding coefficients. Fit: Total inbreeding coefficients.

**Table 2:** Genetic diversity among populations.

Sample Plot		Na	Ne	I	Ho	He	F
Yunnan (China)	Mean	1.667	1.471	0.373	0.079	0.246	0.609
	SE	0.144	0.111	0.078	0.032	0.051	0.115
Baisha (China, Hainan)	Mean	2.048	1.696	0.527	0.111	0.342	0.633
	SE	0.129	0.126	0.073	0.045	0.048	0.108
Qiongzhong (China, Hainan)	Mean	2.238	1.766	0.605	0.148	0.393	0.599
	SE	0.095	0.109	0.052	0.051	0.035	0.117
Wuzhishan (China, Hainan)	Mean	2.143	1.715	0.550	0.140	0.357	0.589
	SE	0.125	0.118	0.070	0.049	0.046	0.116
Ledong (China, Hainan)	Mean	2.048	1.779	0.589	0.196	0.390	0.507
	SE	0.109	0.111	0.062	0.068	0.040	0.144
Dongfang (China, Hainan)	Mean	1.762	1.426	0.389	0.123	0.253	0.543
	SE	0.153	0.130	0.071	0.059	0.048	0.141
Guizhou (China)	Mean	1.952	1.663	0.499	0.138	0.331	0.495
	SE	0.129	0.121	0.073	0.060	0.049	0.142
Baoting (China, Hainan)	Mean	1.905	1.528	0.473	0.151	0.305	0.526
	SE	0.168	0.126	0.072	0.063	0.047	0.148
Jiangxi (China)	Mean	1.286	1.198	0.224	0.111	0.151	0.309
	SE	0.156	0.132	0.074	0.066	0.049	0.195
Guangxi (China)	Mean	1.333	1.237	0.230	0.127	0.159	0.200
	SE	0.126	0.106	0.067	0.067	0.047	0.198
Total	Mean	1.838	1.548	0.446	0.132	0.293	0.532
	SE	0.047	0.039	0.023	0.018	0.015	0.043

**Table 3:** Nei’s genetic distance (upper triangle) and Fst (lower triangle) between populations.

	Yunnan (China)	Baisha (China, Hainan)	Qiongzhong (China, Hainan)	Wuzhishan (China, Hainan)	Ledong (China, Hainan)	Dongfang (China, Hainan)	Guizhou (China)	Baoting (China, Hainan)	Jiangxi (China)	Guangxi (China)
Yunnan (China)	-	0.069	0.112	0.134	0.125	0.284	0.272	0.201	0.241	0.329
Baisha (China, Hainan)	0.076	-	0.038	0.057	0.127	0.183	0.212	0.151	0.219	0.209
Qiongzhong (China, Hainan)	0.099	0.034	-	0.016	0.040	0.163	0.114	0.093	0.169	0.190
Wuzhishan (China, Hainan)	0.120	0.052	0.019	-	0.047	0.177	0.090	0.070	0.139	0.165
Ledong (China, Hainan)	0.165	0.096	0.033	0.039	-	0.176	0.086	0.103	0.189	0.274
Dongfang (China, Hainan)	0.245	0.165	0.134	0.145	0.150	-	0.345	0.212	0.285	0.182
Guizhou (China)	0.214	0.160	0.086	0.075	0.072	0.260	-	0.120	0.148	0.277
Baoting (China, Hainan)	0.192	0.140	0.091	0.076	0.099	0.212	0.122	-	0.211	0.139
Jiangxi (China)	0.307	0.248	0.183	0.175	0.209	0.322	0.212	0.273	-	0.255
Guangxi (China)	0.344	0.223	0.183	0.173	0.241	0.245	0.264	0.203	0.426	-

**Table 4:** Analysis of Molecular Variance of 288 Shanlan upland Rice populations.

Source	DF	SS	MS	Est. Var.	PV (%)	PhiPT
Among Pops	9	435.303	48.367	1.419	8.390	
In Pops	278	4307.269	15.494	15.494	91.609	
Total	288	4742.573		16.913	100%	0.084 (*p* < 0.01)

**Note:** DF: Degree of freedom. SS: Total variance. MS: Mean square variance; EST. Var.: Estimated variance. PV (%): Percent variation. PhiPT: Gene differentiation degree.

**Table 5:** Source and number of Shanlan upland rice.

Origin	Total Number
Jiangxi	3
Guizhou	14
Guangxi	3
Yunnan	3
Baisha (Hainan)	32
Dongfang (Hainan)	12
Qiongzhong (Hainan)	137
Wuzhishan (Hainan)	36
Ledong (Hainan)	36
Baoting (Hainan)	12
Total	288

**Table 6:** 21 pairs of SSR primer information.

No.	Primer	Forward Sequences (5′-3′)	Invert the Sequence (5′-3′)
1	RM7l	agatccatccctgtggagag	gcgaactcgcgttgtaatc
2	OSR28	agcagctatagcttagctgg	actgcacatgagcagagaca
3	RM7102	taggagtgtttagagtgcca	tcggtttgcttatacatcag
4	RM571	ggaggtgaaagcgaatcatg	cctgctgctctttcatcagc
5	RM336	cttacagagaaacggcatcg	gctggtttgtttcaggttcg
6	RM424	tttgtggctcaccagttgag	tggcgcattcatgtcatc
7	RM85	ccaaagatgaaacctggattg	gcacaaggtgagcagtcc
8	RM3331	cctcctccatgagctaatgc	aggaggagcggatttctctc
9	RM567	atcagggaaatcctgaaggg	ggaaggagcaatcaccactg
10	RM551	agcccagactagcatgattg	gaaggcgagaaggatcacag
11	RM331	gaaccagaggacaaaaatgc	catcatacatttgcagccag
12	RM17	tgccctgttattttcttctctc	ggtgatcctttcccatttca
13	RM21	acagtattccgtaggcacgg	gctccatgagggtggtagag
14	RM2l9	cgtcggatgatgtaaagcct	catatcggcattcgcctg
15	RM190	ctttgtctatctcaagacac	ttgcagatgttcttcctgatg
16	RM231	ccagattatttcctgaggtc	cacttgcatagttctgcattg
17	RM253	tccttcaagagtgcaaaacc	gcattgtcatgtcgaagcc
18	RM267	tgcagacatagagaaggaagtg	agcaacagcacaacttgatg
19	RM423	agcacccatgccttatgttg	cctttttcagtagccctccc
20	RM481	tagctagccgattgaatggc	ctccacctcctatgttgttg
21	RM493	tagctccaacaggatcgacc	gtacgtaaacgcggaaggtg

## Data Availability

Data is contained within the article.
